# Identification of Long Non-Coding RNAs Involved in Porcine Fat Deposition Using Two High-Throughput Sequencing Methods

**DOI:** 10.3390/genes12091374

**Published:** 2021-08-31

**Authors:** Yibing Liu, Ying Yu, Hong Ao, Fengxia Zhang, Xitong Zhao, Huatao Liu, Yong Shi, Kai Xing, Chuduan Wang

**Affiliations:** 1 Key Laboratory of Animal Genetics, Breeding and Reproduction, Ministry of Agriculture, National Engineering Laboratory for Animal Breeding, College of Animal Science and Technology, China Agricultural University, Beijing 100193, China; bs20193040371@cau.edu.cn (Y.L.); yuying@cau.edu.cn (Y.Y.); fengxiazhang@cau.edu.cn (F.Z.); zhaoxitong0348@163.com (X.Z.); BS20203040338@cau.edu.cn (H.L.); S20193040546@cau.edu.cn (Y.S.); 2 Institute of Animal Science, Chinese Academy of Agricultural Sciences, Beijing 100193, China; aohong@caas.cn; 3 Animal Science and Technology College, Beijing University of Agriculture, Beijing 102206, China

**Keywords:** lncRNA, fat deposition, pig, RNA-seq

## Abstract

Adipose is an important body tissue in pigs, and fatty traits are critical in pig production. The function of long non-coding RNA (lncRNA) in fat deposition and metabolism has been found in previous studies. In this study, we collected the adipose tissue of six Landrace pigs with contrast backfat thickness (n_high_ = 3, n_low_ = 3), after which we performed strand-specific RNA sequencing (RNA-seq) based on pooling and biological replicate methods. Biological replicate and pooling RNA-seq revealed 1870 and 1618 lncRNAs, respectively. Using edgeR, we determined that 1512 genes and 220 lncRNAs, 2240 genes and 127 lncRNAs were differentially expressed in biological replicate and pooling RNA-seq, respectively. After target gene prediction, we found that *ACSL3* was cis-targeted by lncRNA TCONS-00052400 and could activate the conversion of long-chain fatty acids. In addition, lncRNA TCONS_00041740 cis-regulated gene *ACACB* regulated the rate-limiting enzyme in fatty acid oxidation. Since these genes have necessary functions in fat metabolism, the results imply that the lncRNAs detected in our study may affect backfat deposition in swine through regulation of their target genes. Our study explored the regulation of lncRNA and their target genes in porcine backfat deposition and provided new insights for further investigation of the biological functions of lncRNA.

## 1. Introduction

Fat deposition is an important biological process in pig growth. Fatty traits are critical in pig production and are closely related to pork quality as well as the production efficiency and reproductive traits of pigs [1]. Porcine fat content not only affects the consumers’ choice of pork [2] but is also prone to induce obesity diseases [3], such as type 2 diabetes [4], hypertension, and coronary heart disease [5]. Backfat thickness of swine is a key indicator to judge the fat deposition in commercial pigs. Investigating the measures that decrease backfat deposition is an important approach to effectively accelerate swine genetic improvement. Therefore, it is of great significance to explore the regulators and molecular mechanisms of backfat deposition in pigs. 

Backfat deposition is a complex biological process influenced by multiple genes and epigenetic factors, including long non-coding RNA (lncRNA). In recent years, there have been an increasing number of studies on lncRNA function related to fat development. The regulation of lncRNA on fat has been reported in various species, such as humans, mice, and cattle. For instance, Alvarez et al. [6] used de novo RNA sequencing (RNA-seq) to compare the transcriptomes of different adipose tissues in mice and identified 127 lncRNAs from brown adipose tissue. In addition, they identified lnc-BATE1, which can regulate the growth and thermogenesis of brown adipocytes. Li et al. [7] sequenced preadipocytes and mature adipocytes of Qinchuan cattle and discovered 16 differentially expressed lncRNAs. Among them, lncRNA-ADNCR can inhibit the differentiation of adipocytes by competitively binding to miR-204 with SIRT1. However, there are few studies that focus on the regulation of lncRNA on backfat deposition in pigs [8], especially within one pig breed with extremely high- and low-backfat thickness, and the molecular regulation process of lncRNA in backfat deposition is still unclear. 

With the rapid development of high-throughput sequencing technology, transcriptome sequencing has been widely used in animal studies. Pooling and biological replicate are two conventional methods used in RNA-seq experiments. Pooling RNA-seq refers to mixing individuals in the same treatment group into one pool for sequencing, which is more efficient, less time-consuming, and incurs lower cost when there are many samples [9]. Biological replicate refers to the duplication of samples; in other words, there are multiple samples in the same treatment group used for sequencing, which can eliminate intra-group errors and reduce false-positive rates [10]. These two sequencing methods have their own advantages and disadvantages, but the applicable conditions and accuracy of their results need to be further explored.

In this study, we performed pooling and biological replicate RNA-seq for three pairs of Landrace pigs with contrast phenotypes of backfat thickness. We identified the differentially expressed genes and lncRNAs between the extremely high- and low-backfat pigs. Combining the results of two methods, we obtained the key genes and lncRNAs that affect backfat deposition in the Landrace pigs. Some novel genes and lncRNAs involved in lipid metabolism are provided for decreasing swine backfat deposition in pig breeding. In addition, we also compared the efficiency of the two RNA-seq methods and the accuracy of their results, which provides new ideas for future experimental design in livestock. 

## 2. Materials and Methods

### 2.1. Experimental Animals and Sample Collection

A Landrace female pig resource population was used in this study. We used real-time B-mode ultrasonography (HS1500 convex scanner; Honda Electronics, Toyohashi, Japan) to measure the backfat thickness between the last 3rd and 4th ribs. Combined with pedigree information, we selected three full-sib Landrace pairs with contrast backfat thickness from 132 female Landrace pigs. Three of the pigs with extremely high-backfat thickness formed the high group (BH, 8.88 ± 0.80 mm) and three with extremely low-backfat thickness formed the low group (BL, 3.58 ± 0.39 mm). There was significant difference between the two groups (*p*-value = 1.44 × 10^−3^. The experimental population phenotype and selection criteria were the same as those previously reported [11,12]. The subcutaneous fat tissues of these individuals were collected after slaughter and then immediately stored in liquid nitrogen until use. 

### 2.2. Pooling RNA-seq and Biological Replicate RNA-seq Designs

**Figure 1:**
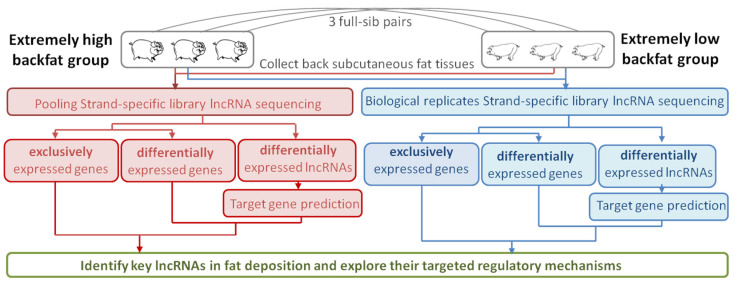
Experimental design of the current study.

In our study, three pairs of full-sib Landrace pigs with either extremely high- or low-backfat thickness were taken as paired samples. In pooling strand-specific library RNA-seq, six pigs were divided into two separate pools according to their contrast backfat thickness, among which one pool comprised pigs with extremely high backfat (BH) and the other comprised pigs with extremely low backfat (BL). Then, pooling RNA-seq was performed using these two groups. In biological replicate strand-specific library RNA-seq, there were three independent individuals with similar phenotypes in both BH and BL groups. We identified the differentially expressed genes and lncRNAs in the two-method results and then analyzed their function in fat development. We also explored the specific expressed genes and lncRNAs in different phenotype groups based on the two RNA-seq. The experimental design is shown in Figure 1.

### 2.3. RNA Extraction and Sequencing

We used a total RNA extraction kit (Bioteke Corporation, Wuxi, China) to extract the total RNAs of all the samples, according to the manufacturer’s recommendations, and the quality of the extract was checked by a 2100 Bioanalyzer (Agilent Technologies, Santa Clara, CA, USA). After quality control, a library was prepared using the total RNAs. The sequencing was performed by Biomarker Technologies, Inc. (Rohnert Park, CA, USA) in an Illumina Hiseq 2500 sequencer (Illumina, Inc., San Diego, CA, USA). The raw data obtained from the sequencing were trimmed using Trimmomatic software [13]. Clean data were achieved after deleting the adapter and low-quality reads (Phred quality score ≤ 10). Using FastQC (v 0.11.8) [14] to check the quality of the paired-end reads of all the six samples. We then used a NGSQC Toolkit (v 2.3.3) [15] to perform quality inspection and data filtering of the high-throughput sequencing data. Only good quality trimmed reads were considered for downstream analysis.

### 2.4. Mapping and Assembly of Sequenced RNA Reads

The pig reference genome (Sscrofa 11.1) and annotation files were downloaded from the Ensembl database (http://asia.ensembl.org/Sus_scrofa/Info/Index, accessed on 20 June 2021) and indexed with Bowtie2 (v 2.3.0) [16]. Clean reads were aligned with the reference genome using TopHat2 (v 2.1.1) [17] alignment program. Software Cufflinks (v 2.2.1) [18] was used to assemble the mapped reads and determine their abundance in all the samples, keeping all parameters as default. Cuffmerge, a Cufflinks suite tool, was used to merge samples in both pooling and biological replicate RNA-seq, then obtain the merged gene transfer format (gtf) file for further downstream analysis.

### 2.5. LncRNA Filtering Pipeline

High stringency was used to filter out putative lncRNAs from the RNA-seq assembled transcripts. The information in the assembled transcripts from the Cuffmerge results included the class code of each transcript, such as ‘=’, ‘’., ‘c’, ‘j’, ‘e’, ‘i’, ‘o’, ‘p’, ‘r’, ‘u’, ‘x’, and ‘s’. The transcripts with non-coding potential class codes of ‘j’, ‘i’, ‘o’, ‘u’, ‘x’, and ‘=’ were reserved [19] and all others were removed. Transcripts with less than 200 nucleotides and less than 2 exons were also deleted. Filtered transcripts were subjected to three of the most frequently used tools for identifying long non-coding RNAs, two of which were the Coding Potential Calculator (CPC) [20] and Coding-Non-Coding-Index (CNCI) [21]. Common transcripts from the results of these two tools were then scanned against the Pfam database to discover any probable protein domains. We used the hmmer-3.2.1 (Howard Hughes Medical Institute, San Francisco, MD, USA) to identify the transcripts translated in all six possible frames with homologs, and the transcripts matched to the Pfam hit were excluded with an E-value < 1 × 10^−5^ [22]. Then we used the BLASTX program to detect the similarity between the transcripts and the known protein in NCBI NR database. The transcripts with an E-value < 1 × 10^−5^ were filtered out [23]. Similar protocols have been used in previous studies to identify lncRNAs [8].

### 2.6. Identification of Differentially and Specific Expressed lncRNA and mRNA

We used htseq software (v 0.7.2) [24] to count the reads of genes and lncRNAs, and further screened them by their expression level in extremely high-or low-backfat thickness group. The edgeR package [25] was used to determine the expression difference between the two groups using an over-dispersed Poisson model. We used false discovery rate (FDR) ≤ 0.05 and |Fold Change| > 2 as the threshold to identify significantly differentially expressed lncRNAs (DELs) and differentially expressed genes (DEGs). Heatmap clustering analysis of all differentially expressed lncRNAs and messenger RNAs (mRNAs) was calculated using the heatmap [26] R package. We utilized fragments per kilobase of transcript per million mapped reads (FPKM) ≥ 0.1 as the criteria to ensure expression. In biological replicate RNA-seq, we calculated the average FPKM of three samples in the same group. LncRNAs expressed at marginal levels (FPKM/average FPKM < 0.1) were removed. If the transcripts were expressed only in one group but not in the other group, we considered them to be specific expressed lncRNAs (SELs) and specific expressed genes (SEGs).

### 2.7. Gene Ontology and KEGG Pathway Functional Annotations

Gene Ontology (GO, http://www.geneontology.org, accessed on 20 June 2021) [27] is the international standard classification of gene function. It classifies the function of genes along three aspects. The Kyoto Encyclopedia of Genes and Genomes (KEGG, http://www.genome.jp/kegg, accessed on 20 June 2021) [28] is a genomic information database that helps researchers to study genes and expression information as a whole network. The functional annotation of the DEGs in this study based on GO and KEGG was completed using DAVID (v 6.8) [29] and KOBAS 3.0 [30], respectively. The GO terms and KEGG pathways with *p*-values < 0.05 were considered as significantly enriched, and the results were plotted using the ggplot2 [31] R package.

### 2.8. Target Genes Prediction and Functional Annotations

To investigate the function of the differentially expressed lncRNAs, we searched their nearby genes and considered them as potential targets of lncRNAs. It has been reported that the main function of lncRNA is to regulate protein-coding genes through cis- and trans-regulation [32,33]. The general method of predicting lncRNA target genes is to search upstream or downstream to identify nearby protein-coding regions, termed as cis-regulating target genes [34]. It has been reported that lncRNA can regulate coding genes around 10 to 500 kb up and downstream [35]. Bedtools (v2.25.0) [36] was used to search neighborhood genes around 100 kb upstream and downstream of the DELs. In addition, if the expression patterns of lncRNA and mRNA show a highly positive or negative correlation, their functions may be highly correlated [37]. Therefore, we examined the Spearman’s rank correlation between the expression levels of the DELs and DEGs to predict their targeting relationship.

### 2.9. Statistical Analysis

All data were reported as mean ± standard error of mean. In the edgeR package, we used the qCML (quantile-adjusted conditional maximum likelihood) method to estimate the dispersion and the exactTest function for differential expression analysis; the FDR method was used to adjust the p values. An FDR < 0.05 was considered significant.

### 2.10. Validation of Differentially Expressed Genes and lncRNAs

Total RNA was extracted from the fat tissues and converted into cDNA using the TaKaRa PrimeScript™RT reagent Kit (Thermo Fisher Scientific Inc, Waltham, MA, USA), following the manufacturer’s protocol. cDNA samples were analyzed with real-time reverse transcriptase (RT)-PCR using the Light Cycler^®^ 480 Real-Time PCR System (Roche, CA, USA). Primers used for quantification were designed by Primer-Premier (v6.0) and Primer-BLAST on the NCBI website (https://www.ncbi.nlm.nih.gov/tools/primer-blast/, accessed on 5 June 2021). RT-PCR reactions were performed in a final volume of 20 μl with the Roche SYBR Green PCR Kit (Roche), according to the manufacturer’s instructions. Pig GAPDH was used as an internal standard to correct the cDNA input. Triplicate RT-qPCRs were performed for each cDNA and the average Ct was used for further analysis. The PCR program was run at 95 °C for 3 min and then 45 cycles of 95 °C for 10 s, 60 °C for 30 s, and 72 °C for 1 min. The melting curve was then run for 65–95 °C. Relative quantification values were calculated using the 2^−ΔΔCt^ method.

## 3. Results

### 3.1. Overview of lncRNA Sequencing Data Based on Pooling and Biological Replicate RNA-seq 

In the pooling RNA-seq, a total of 15.53 and 14.30 Gb of clean data were separately generated in the extremely high- and low-backfat thickness groups. The guanine-cytosine (GC) contents of the BH and BL group were 51.38% and 49.37%, respectively, and their Q30 were 92.53% and 92.62%, respectively. Furthermore, the results of the biological replicate RNA-seq were 16.20, 16.32, 17.34, 16.24, 16.07, and 16.50 Gb in the six samples. The average of the GC content was between 50.71% and 55.55%, and Q30 ranged from 93.32% to 94.27%. These results showed that the quality of the two libraries of pooling RNA-seq and the six libraries of biological replicate RNA-seq was suitable for subsequent data analysis.

Next, the clean reads were aligned to the reference genome (Sus scrofa 11.1) using Tophat v2.1.1. In pooling RNA-seq, more than 77.82% of the clean reads were uniq mapped. In all samples of biological replicate RNA-seq, the uniq mapped ratios were larger than 83.77%. The summary of the sequencing data is shown in Table 1.

### 3.2. Identification and Feature Analysis of Putative lncRNAs in Landrace Pig Backfat

**Figure 2:**
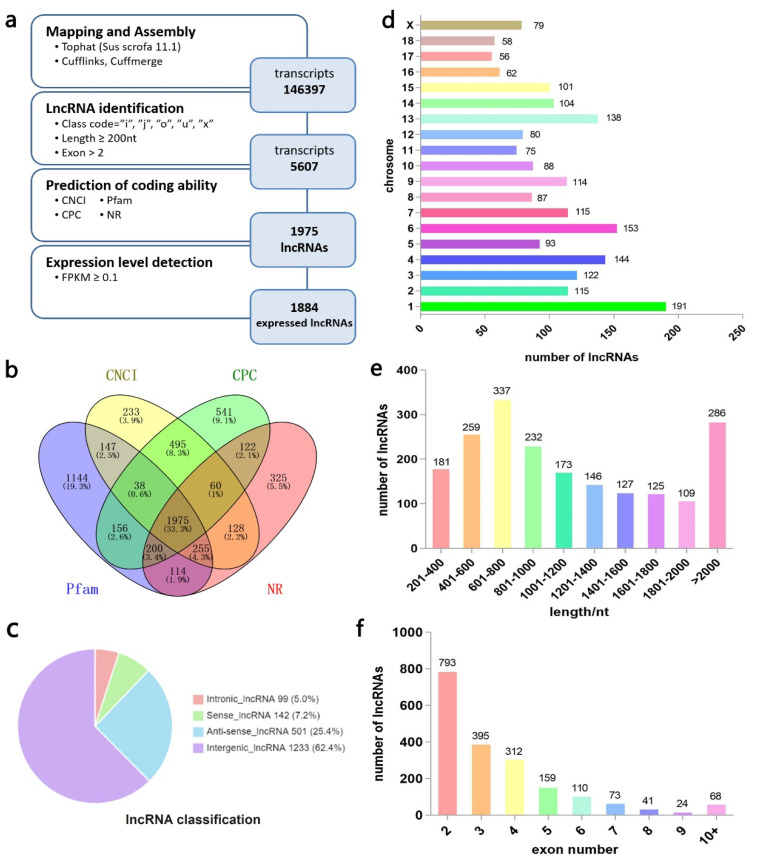
Summary of putative long non-coding RNA (lncRNA) characteristics in Landrace pig backfat. (**a**) Identification pipeline of lncRNAs; (**b**) Venn diagram of identified lncRNAs from the Coding Potential Calculator (CPC), Coding-Non-Coding Index (CNCI), Pfam, and NR database; (**c**) Type and number of putative lncRNAs; (**d**) Chromosome distribution of lncRNAs; (**e**) Length distribution of lncRNAs; (**f**) Exon number distribution of lncRNAs.

After using cufflinks and cuffmerge, we obtain a combined GTF file containing 146,397 transcripts. Then we followed stringent criteria and created a pipeline to identify lncRNAs, as shown in Figure 2a. Finally, we identified 1975 lncRNAs (Figure 2b). Among them, 334 were known transcripts and 1641 were novel transcripts, including 1233 lincRNAs (62.4%), 501 anti-sense lncRNAs (25.4%), 142 sense lncRNAs (7.2%), and 99 intronic lncRNAs (5.0%) (Figure 2c). These 1975 lncRNAs were distributed throughout all pig chromosomes, but chromosome 1 contained the most lncRNAs (Figure 2d). The majority of the lncRNA length was between 601 and 800 nt, and lncRNA with two exons were the most common, as shown in Figure 2e,f.

### 3.3. Expression Analysis of Differentially and Specific Expressed Genes and lncRNAs in Extremely High- or Low-Backfat Pigs Based on Pooling RNA-Seq

**Figure 3:**
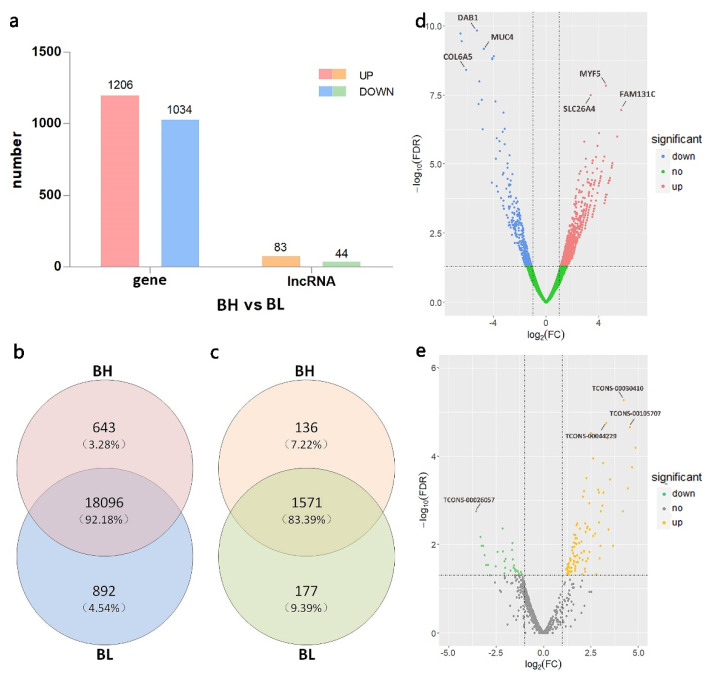
Expression of genes and long non-coding RNAs (lncRNAs) in extremely high- and low-backfat pigs based on the pooling RNA sequencing (RNA-seq). (**a**) Number of differentially expressed lncRNAs (DELs) and differentially expressed genes (DEGs) between the BH and BL groups; (**b**) Venn diagram of the genes from each group; (**c**) Venn diagram of the lncRNAs from each group; (**d**) Volcano plot of DEGs. The Y-axis is the value of −log10 (false discover rate [FDR]) and the X-axis is the value of log2(FC). The two threshold lines show the standard of FDR = 0.05 and FC = 2; (**e**) Volcano plot of DELs. We have annotated the most significant DEGs and DELs in the volcano.

In the pooling RNA-seq, 2240 genes and 127 lncRNAs (Table S1) were differentially expressed between the two groups, of which 1206 genes were up-regulated in the BH group compared to those in the BL group and 1034 were down-regulated (Figure 3a,d). The expression of 83 lncRNAs was up-regulated in the BH group, and 44 lncRNAs were down-regulated (Figure 3e).

In addition, to further explore the difference in gene expression between the extremely high- and low-backfat thickness groups, we analyzed the specific expressed genes and lncRNAs in the two groups. There were 643 SEGs and 136 SELs in the BH group and 892 DEGs and 177 DELs in the BL group in pooling RNA-seq (Figure 3b,c).

### 3.4. Expression Analysis of Differentially and Specific Expressed Genes and lncRNAs in Extremely High- or Low-Backfat Pigs Based on Biological Replicate RNA-seq

**Figure 4:**
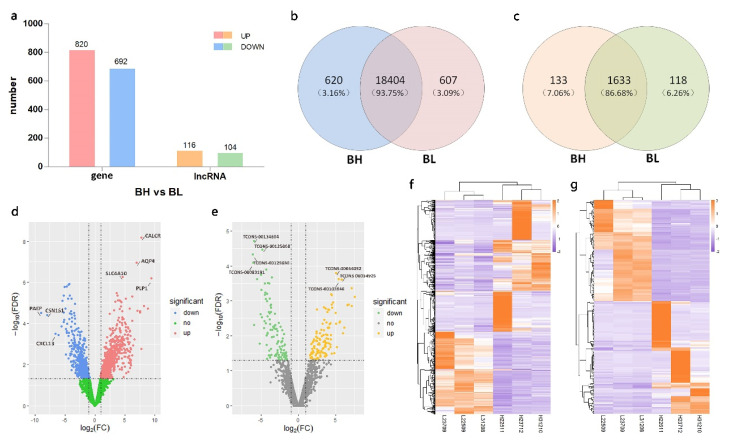
Expression of genes and long non-coding RNAs (lncRNAs) in extremely high- and low- backfat pigs based on biological replicate RNA sequencing (RNA-seq). (**a**) Number of differentially expressed lncRNAs (DELs) and differentially expressed genes (DEGs) between the BH and BL groups; (**b**) Venn diagram of genes from each group; (**c**) Venn diagram of lncRNAs from each group; (**d**) Volcano plot of DEGs; (**e**) Volcano plot of DELs; (**f**) Heatmap of DEGs in six samples. The color scale indicates the FPKM values. Orange indicates high expression and purple indicates low expression; (**g**) Heatmap of DELs in six samples. We have annotated the most significant DEGs and DELs in the volcano.

In the biological replicate RNA-seq, we performed the same analysis as that used for the pooling RNA-seq. We found that 1512 genes and 220 lncRNAs (Table S1) were differentially expressed in the two groups, of which 820 genes were up-regulated in the BH group compared to those in the BL group and 692 were down-regulated (Figure 4a,d). There were 116 lncRNAs with up-regulated expression levels in the BH group, and 104 lncRNAs were down-regulated (Figure 4e). Next, we used the 1512 differentially expressed genes and 220 differentially expressed lncRNAs to perform a clustering analysis. As shown in the heatmap, the expression patterns of the samples in the BH group were distinguished from their expression patterns in the BL group, both for the DEGs (Figure 4f) and DELs (Figure 4g).

Moreover, we identified 620 genes and 133 lncRNAs specific expressed in the BH group and 607 genes and 118 lncRNAs specific expressed in the BL group based on the result of biological replicate RNA-seq (Figure 4b,c).

### 3.5. GO and KEGG Functional Enrichment Analysis of DEGs and SEGs Based on Pooling and Biological Replicate RNA-Seq

**Figure 5:**
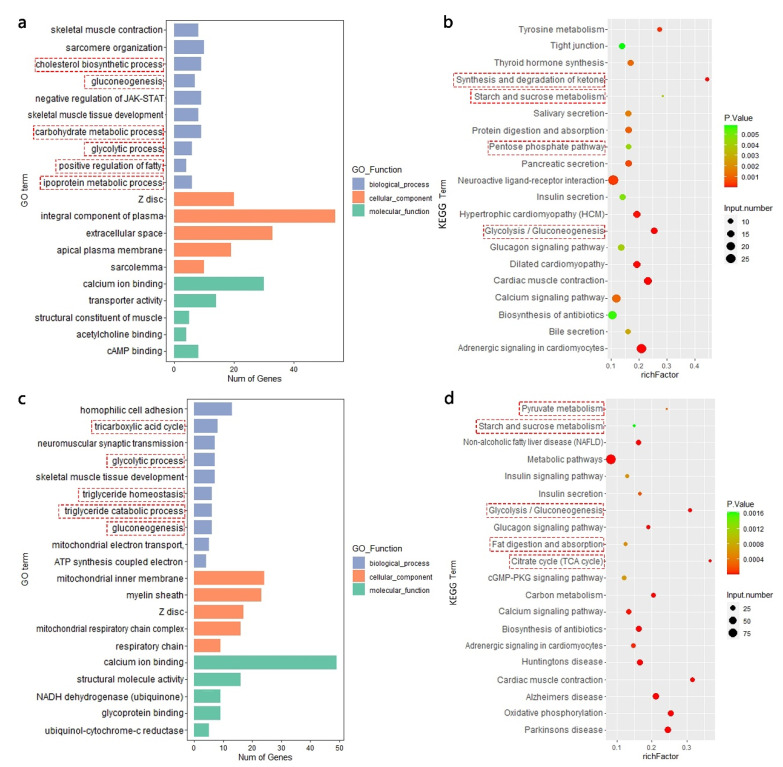
Functional enrichment analyses of differentially expressed genes (DEGs) and specific expressed genes (SEGs) based on pooling and biological replicate RNA sequencing (RNA-seq). (**a**) Gene Ontology (GO) annotation of DEGs in pooling RNA-seq; (**b**) Kyoto Encyclopedia of Genes and Genomes (KEGG) pathway analysis of DEGs in pooling RNA-seq; (**c**) GO annotation of DEGs in biological replicate RNA-seq; (**d**) KEGG pathway analysis of DEGs in biological replicate RNA-seq. The red boxes indicate the GO terms and KEGG pathways related to glucose and lipoid metabolism. (**a**,**c**) show the 10 most significant (FDR < 0.05) terms in the Biological Process (BP) class and the 5 most significant terms in the Cellular Component (CC) and Molecular Function (MF) classes. (**b**,**d**) show the most significant 20 pathways in the KEGG results.

We used the differentially expressed genes detected in the pooling and biological replicate RNA-seq methods to perform functional enrichment analysis, respectively. The Gene Ontology results of DEGs based on the pooling RNA-seq are shown in Figure 5a. We focused on several terms, including ‘positive regulation of fatty acid biosynthetic process’, ‘lipoprotein metabolic process’, and ‘carbohydrate metabolic process’. In addition, DEGs were also enriched in some glucose metabolism pathways such as ‘gluconeogenesis’ and ‘glycolytic process’. Simultaneously, these DEGs also underwent a KEGG analysis to annotate their functions. As shown in Figure 5b, in the pooling RNA-seq, we found that ‘Synthesis and degradation of ketone bodies’, ‘Pentose phosphate pathway’, and ‘Starch and sucrose metabolism’ were enriched, all of which are important in the glucose metabolism program. All the terms that were considered important are highlighted by a red frame in Figure 5.

In the biological replicate RNA-seq, a total of 977 DEGs were annotated in GO terms. We found many terms directly related to fat development and metabolism, especially in the BP class, including ‘tricarboxylic acid cycle’, ‘triglyceride homeostasis’, and ‘triglyceride catabolic process’. Furthermore, there were four mRNAs enriched to ‘positive regulation of triglyceride catabolic process’. This GO term was also significant but not within the top ten; therefore, it is not displayed in Figure 5c. Moreover, the KEGG results of DEGs based on biological replicate RNA-seq are shown in Figure 5d. Some pathways related to lipoid metabolism were significantly enriched, including ‘Citrate cycle (TCA cycle)’ and ‘Fat digestion and absorption’, which were not previously enriched. We also circled the important pathways with a red frame in Figure 5.

In addition, we used the specific expressed genes from the pooling and biological replicate RNA-seq to perform the functional enrichment analysis, respectively. These results are shown in Figure S1. There are only few KEGG pathways related to glucose and lipoid metabolism, most of which are involved in the SEGs in the BH group of biological replicate RNA-seq (Figure S1b). These pathways include ‘Fat digestion and absorption’, ‘Synthesis and degradation of ketone bodies’, and ‘Pentose phosphate pathway’. We believe that the other GO terms and KEGG pathways are not as necessary for fat deposition. 

### 3.6. Comparison of Pooling and Biological Replicate RNA-seq

We compared the library construction quality of the two sequencing methods (Table 1). We found that the amount of clean data in biological replicate RNA-seq was larger and the average of GC content and Q30 of these samples was higher than those of pooling RNA-seq. In addition, we aligned clean reads with the reference genome and compared them with those of pooling RNA-seq; the percentages of uniq mapped reads were more in biological replicate RNA-seq than in pooling RNA-seq.

**Figure 6:**
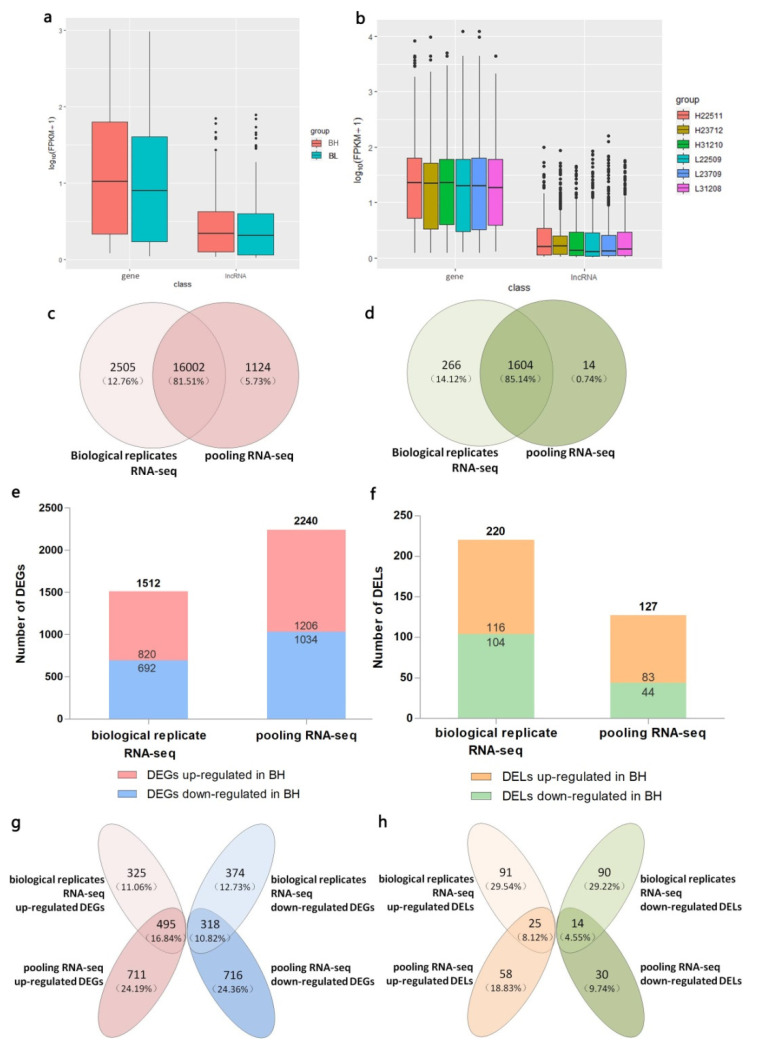
Comparison of pooling and biological replicate RNA sequencing (RNA-seq). (**a**) Comparison of expression levels between the genes and lncRNAs in two groups of pooling RNA-seq. The value of log10 (fragments per kilobase of transcript per million mapped reads [FPKM] +1) is plotted on the *Y*-axis; (**b**) Comparison of the expression levels between the genes and lncRNAs in six samples of biological replicate RNA-seq; (**c**) Venn diagram of unique genes between two sequencing methods; (**d**) Venn diagram of unique lncRNAs between two sequencing methods; (**e**) Number of differentially expressed genes (DEGs) between BH and BL groups of two sequencing methods; (**f**) Number of differentially expressed lncRNAs (DELs) between BH and BL groups of two sequencing methods; (**g**) Venn diagram of DEGs of two sequencing methods; (**h**) Venn diagram of DELs of two sequencing methods.

In the present study, we compared the expression of the putative lncRNAs with the genes. We found that the lncRNAs tended to be expressed at a lower level than the protein-coding genes in both the pooling and biological replicate RNA-seq (Figure 6a,b). A total of 19,631 genes and 1975 lncRNAs were identified, and within these, 16,002 genes and 1604 lncRNAs were jointly identified in both sequencing results. Further, 124 genes and 14 lncRNAs were uniquely detected in the pooling RNA-seq. In the biological replicate RNA-seq, there were 2505 unique genes and 266 unique lncRNAs (Figure 6c,d). For differentially expressed genes and lncRNAs (Figure 3 and Figure 4), we found 2240 DEGs in pooling RNA-seq, which were more than 1512 DEGs in biological replicate RNA-seq (Figure 6e). However, only 127 DELs were found in pooling RNA-seq, which were less than 220 DELs in biological replicate RNA-seq (Figure 6f). There were 813 DEGs and 39 DELs identified in both two sequencing methods (Figure 6g,h).

In the functional enrichment analysis of DEGs (Figure 5), both biological replicate and pooling RNA-seq enriched several important GO terms and KEGG pathways related to glucose metabolism, and the results of the two sequencing methods partially overlapped. For example, GO terms such as ‘gluconeogenesis’ and ‘glycolytic process’ in the result of pooling RNA-seq were same as those in biological replicate RNA-seq. KEGG pathway ‘Glycolysis/Gluconeogenesis’ also existed in the 20 most significant pathways of both methods. The unique genes of the two sequencing methods were then subjected to GO and KEGG functional enrichment analyses. None of the GO terms or KEGG pathways were related to lipid metabolism in this analysis. These results are shown in Figure S2.

### 3.7. Target Gene Prediction of Differentially Expressed lncRNAs 

To further understand the potential function of lncRNA in fat deposition, we predicted the target genes of the lncRNAs. To increase the reliability of our results, we selected the DELs that were shared by the two sequencing methods and then predicted their target genes. We searched the nearby coding genes around 100 kb up- and downstream from the 39 DELs and found 76 pairs of cis-regulatory relationships between 39 DELs and 67 genes (Table S3). 

**Figure 7:**
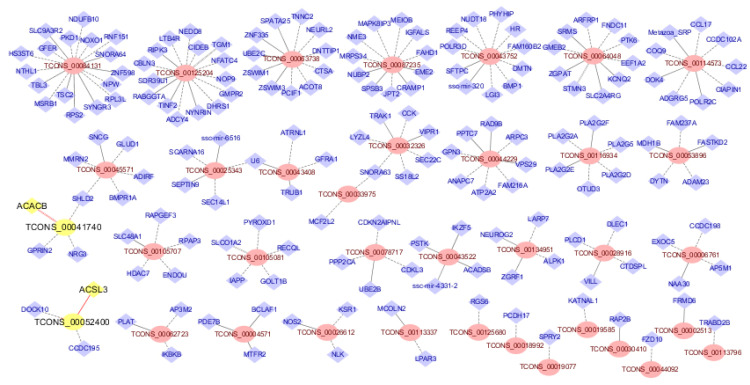
Network of regulatory relationships between differentially expressed long non-coding RNAs (DELs) and their target genes. DELs are indicated in red ovals; target genes are indicated in blue diamonds; the important long non-coding RNAs and genes are highlighted with yellow nodes and red labels. Cis-regulation is represented by a solid line, trans-regulation is represented by a dotted line, and dual-regulation is represented by a double line.

In addition, we conducted a Spearman’s rank correlation analysis between the expression of the DELs and DEGs. The expression levels of 116 DEGs were significantly correlated with the 39 DELs (correlation coefficient > 0.934, *p* < 0.05). They were also considered as potential target genes of these DELs. We used 39 DELs and their target genes to draw a picture of the regulatory network to investigate the relationship between them (Figure 7, Table S3). We highlighted the lncRNAs and their target genes for further analysis with yellow nodes and red labels.

### 3.8. Validation of DEGs and DELs through qRT-PCR

**Figure 8:**
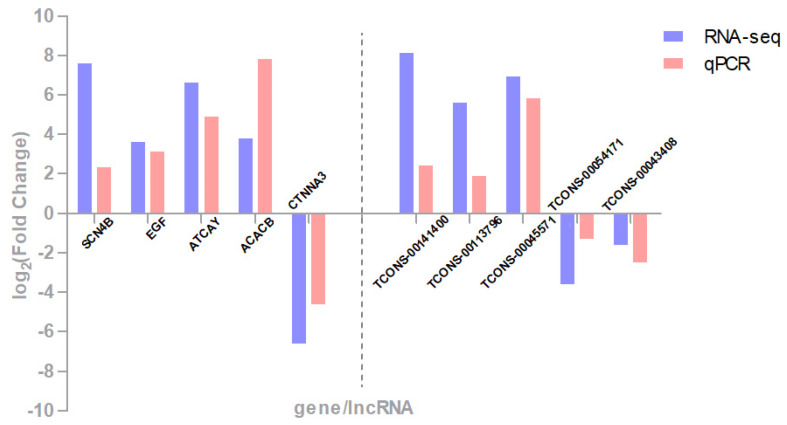
Validation of DEGs and DELs through qRT-PCR.

We randomly selected 5 DEGs (*SCN4B*, *EGF*, *ATCAY*, *ACACB*, and *CTNNA3*) and 5 DELs (TCONS-00141400, TCONS-0113796, TCONS-00045571, TCONS-00054171, and TCONS-00043408) to perform qPCR. As shown in Figure 8, the results of qPCR verified RNA-seq analysis and indicated the quality of the sequencing data.

## 4. Discussion

In most of the previous studies, researchers identify key lncRNAs between different breeds of pigs [38,39]. However, our research used contrast backfat thickness individuals within same the breed as the samples to eliminate inter-species differences. At the same time, we selected full-sib pairs to minimize the influence of genetic background. We also used pooling and biological replicate RNA sequencing to identify critical lncRNAs involved in porcine backfat deposition. In this study, we performed pooling and biological replicate RNA-seq on the same samples, which can compare the accuracy and reliability of the two sequencing methods. In addition, when we analyzed the function of critical lncRNAs involved in porcine backfat deposition, we used the lncRNA shared by the two RNA-seq methods, which also increased the reliability of our results.

In this study, biological replicate RNA-seq detected more genes than pooling RNA-seq did. However, in the function annotation, the unique genes of both sequencing methods did not enrich the GO terms and KEGG pathways related to glucose and lipid metabolism. The above results indicate that the overlap genes of pooling and biological replicate RNA-seq may have a considerable effect on fat deposition in Landrace pigs. Next, compared with pooling RNA-seq, biological replicate RNA-seq detected more lncRNAs and DELs. The low expression of lncRNAs could be covered up [10]. This means biological replicate RNA-seq is more efficient for mining lncRNAs or other type RNAs.

Studies have shown that the expression level of lncRNAs is usually lower than that of protein-coding genes [40], we found that the expression of lncRNA was much lower than that of the gene. We identified 18,507 genes and 1870 lncRNAs in pooling RNA-seq, and 17,126 genes and 1618 lncRNAs in biological replicate RNA-seq. Our results are similar to those in several previously published articles on adipose tissue transcriptome sequencing. For example, Miao et al. [41] identified 4910 lncRNAs, 119 of which were differentially expressed in the intramuscular fat tissue of Jinhua and Changbai pigs. The results for the functional enrichment of the DEGs showed that some genes were enriched to pathways related to glucose and lipid metabolism. In addition, these genes were confirmed to be related to lipid metabolism in previous studies, including *APOA1* [42] and *STARD3* [43]. Chen et al. [8] identified 581 putative lincRNAs related to pig muscle growth and fat deposition, and their target genes were involved in fat deposition-related processes, such as the lipid metabolic process and fatty acid degradation. The KEGG results showed that the meaningful pathways were mostly concentrated on glucose metabolism. Some of these pathways, such as ‘Glycolysis/Gluconeogenesis’, were also found in a previous study by our team [11]. Although they are not directly related to lipid synthesis and metabolism, glucose and lipids can change into each other and participate together in the tricarboxylic acid cycle. Therefore, the process of glucose metabolism can have an indirect effect on lipid metabolism. 

As a kind of non-coding RNA, the main role of lncRNA is to regulate their target genes: cis-regulating nearby protein-coding genes and trans-regulating distant protein-coding genes. We determined 67 cis-target genes and 116 trans-target genes regulated by DELs. The two target genes we focused on were *ACACB* and *ACSL3*, both of which have been found to be related to lipid metabolism in other studies. *ACLS3* (acyl-CoA synthetase long chain family member 3) is located 64 kb downstream of TCONS-00052400. At the beginning of fatty acid metabolism, long-chain acyl-CoA synthetase (*ACSL*) can activate the conversion of long-chain fatty acids to fatty acyl-CoA [44]. The *ACSL* family contains five different isoforms, including *ACSL3*, which play different roles in lipid metabolism. Some previous studies showed that knockdown of *ACSL3* may significantly reduce the activity of several lipid-producing transcription factors such as peroxisome proliferator-activated receptor-γ and sugar response element binding protein, and then regulate the fat production process in the liver [45]. Furthermore, *ACSL3* can also affect the secretion of very low-density lipoproteins (VLDL) by promoting the synthesis of lecithin [46]. *ACACB* (acetyl-CoA carboxylase beta) is a differentially expressed gene that is cis-regulated by the lncRNA TCONS-00041740, which is located on the antisense strand of *ACACB*. Moreover, in the KEGG enrichment analysis, *ACACB* was involved in the ‘pyruvate metabolism’ pathway. Based on previous studies, we knew that *ACACB* is the rate-limiting enzyme in fatty acid oxidation [47]. Moreover, in *ACACB* knockout mice, continuous fatty acid oxidation increases insulin sensitivity, and feeding them a high fat/high carbohydrate diet is more likely to cause obesity and diabetes [48]. In a previous study by Li et al. [49], *ACACB* was shown to be a marker gene for childhood obesity.

Some genes were specific expressed in one of the two groups. For example, *PLCB2* (phospholipase C beta 2) was specific expressed in the BL group. Phospholipase C is a class of glycerol phospholipid hydrolases, hydrolyzing the glycerol phosphate C3 site [50]. The protein encoded by *PLCB2* is a phosphodiesterase that catalyzes the hydrolysis of phosphatidylinositol 4, 5-bisphosphate to the secondary messengers inositol 1, 4, 5-trisphosphate (IP3) and diacylglycerol. In addition, the gene *PLA2G12B* was specific expressed in the BH group. *PLA2G12B* (phospholipase A2 group XIIB) is encoded by this gene and belongs to the phospholipase A2 (*PLA2*) group of enzymes, which plays a role in lipid hydrolysis by releasing free fatty acids and lysophospholipids [51]. Studies have shown that a reduction of *PLA2G12B* decreases the amount of serum triglyceride (TG)-rich VLDL particles secreted by the liver, resulting in a reduction in TG content [52]. *PLA2G12B* can also participate in the pathogenesis of idiopathic membranous nephropathy (iMN) by regulating lipid metabolism [53]. In a previous study by Guan et al. [54], *PLA2G12B*-null mice had obvious accumulation of large lipid droplets in the liver, displaying the fatty liver phenotype. These results indicate that the genes specific expressed in the high or low backfat groups may also have a certain regulatory effect on lipid metabolism.

During pig growth, the excessive development of adipose tissue leads to an excessive accumulation of lipids, which affects the carcass quality of pigs. Overweight pigs show low lean meat rates, low feed conversion rates, and slow growth [55], while overweight sows may experience dystocia and postpartum disease [56]. Numerous studies have shown that pigs have many similarities with humans in anatomical structure, physiological metabolism, and disease mechanisms [57,58]. Therefore, pigs have many advantages as an animal model for human disease research, especially related to obesity [59,60]. Pig backfat thickness can directly reflect their body fat content. Our study obtained differentially expressed lncRNAs between pigs with extremely high- and low- backfat thickness, providing a novel approach for the use of lncRNAs and their target genes to screen low backfat pigs in future breeding work, thereby further improving fat deposition traits in pigs. However, further genetic experiments are still needed to validate the association of the lncRNA and mRNA functions presented in this study.

## 5. Conclusions

In this study, we identified 1512 DEGs and 220 DELs between pigs with extremely high- and low- fat deposition traits in biological replicate RNA-seq, and 2240 DEGs and 127 DELs in pooling RNA-seq, respectively; the former revealed more genes, but the two methods were similar in terms of gene functional enrichment. After the analysis of potential cis- and trans- target genes, we found 183 genes that could be regulated by DELs. Through further functional analysis, we found that two pairs of potential targeting relationships between lncRNAs and genes, TCONS-00041740 to *ACACB*, and TCONS-00052400 to *ACSL3*, may have an effect on fat deposition. These results can provide useful information for understanding the regulation of fat deposition by lncRNA in pigs. 

## Figures and Tables

**Table 1:** Summary of reads after quality control and mapping to the reference genome.

	Pooling RNA Sequencing		Biological Replicate RNA Sequencing					
Sample ID	BH	BL	H22511	H23712	H31210	L22509	L23709	L31208
Clean Data	15,526,646,169	14,298,214,979	16,202,102,282	16,317,045,132	17,343,934,922	16,244,957,328	16,074,210,300	16,498,461,484
GC (%)	51.38	49.37	50.71	54.34	55.24	50.89	55.55	51.63
Q30 (%)	92.53	92.62	94.27	93.32	93.71	93.76	93.84	93.76
Total Reads	106,068,646	97,723,146	108,162,730	109,081,084	115,964,182	109,091,500	107,499,706	110,216,006
Mapped Reads	84,998,524	79,213,694	103,261,178	99,528,016	106,878,278	101,789,248	96,471,336	103,862,050
	(80.14%)	(81. 06%)	(95.47%)	(91.24%)	(92.16%)	(93.31%)	(89.74%)	(94.23%)
Uniq Mapped Reads	82,541,284	77,406,164	96,976,910	93,090,379	97,146,560	97,935,676	92,128,163	98,096,189
	(77.82%)	(79.21%)	(89.66%)	(85.34%)	(83.77%)	(89.77%)	(85.70%)	(89. 00%)
Multiple Mapped Reads	2,457,240	1,807,530	6,284,268	6,437,637	9,731,718	3,853,572	4,343,173	5,765,861
	(2.32%)	(1.85%)	(5.81%)	(5.90%)	(8.39%)	(3.53%)	(4. 04%)	(5.23%)

Guanine-cytosine (GC) (%) is the percentage of G and C bases in the total nucleotides; Q30 (%) is the percentage of the bases’ mass greater than or equal to Q30 in the clean data; Total Reads is the number of clean reads; Uniq Mapped Reads is the number and percentage of reads that were mapped to a unique position in the reference genome in the clean reads; Multiple Mapped Reads is the number and percentage of reads that were mapped to multiple positions in the reference genome in the clean reads.

## Data Availability

The deep sequencing data of total RNA were submitted to NCBI Sequence Read Archive (SRA) with accession number Bioproject: PRJNA6600160.

## Supplementary Materials

The following are available online at https://www.mdpi.com/article/10.3390/genes12091374/s1, Table S1: List of differentially expressed genes and lncRNAs in two high-throughput sequencing methods, Table S2: List of cis- and trans- target genes of common DELs, Table S3: List of differentially expressed lncRNAs and their target genes, Figure S1: Functional enrichment analysis of specific expressed genes in porcine fat, Figure S2: Functional enrichment analysis of unique genes in pooling and biological replicate RNA sequencing.
