# Supplementary material for: Identification of Long Non-Coding RNAs Involved in Porcine Fat Deposition Using Two High-Throughput Sequencing Methods
Source: Genes (Basel). 2021 Aug 31;12(9):1374. doi: 10.3390/genes12091374 (PMC8467702; doi:10.3390/genes12091374)

Additional file 5

Figure S2 Functional enrichment analysis of unique expressed genes of biological replicate and pooling RNA sequencing (RNA-seq) in porcine fat

(a) Gene Ontology (GO) annotation of unique genes in pooling RNA-seq; (b) Kyoto Encyclopedia of Genes and Genomes (KEGG) pathway analysis of unique genes in pooling RNA-seq; (c) GO annotation of unique genes in biological replicate RNA-seq; (d) KEGG pathway analysis of unique genes in biological replicate RNA-seq.

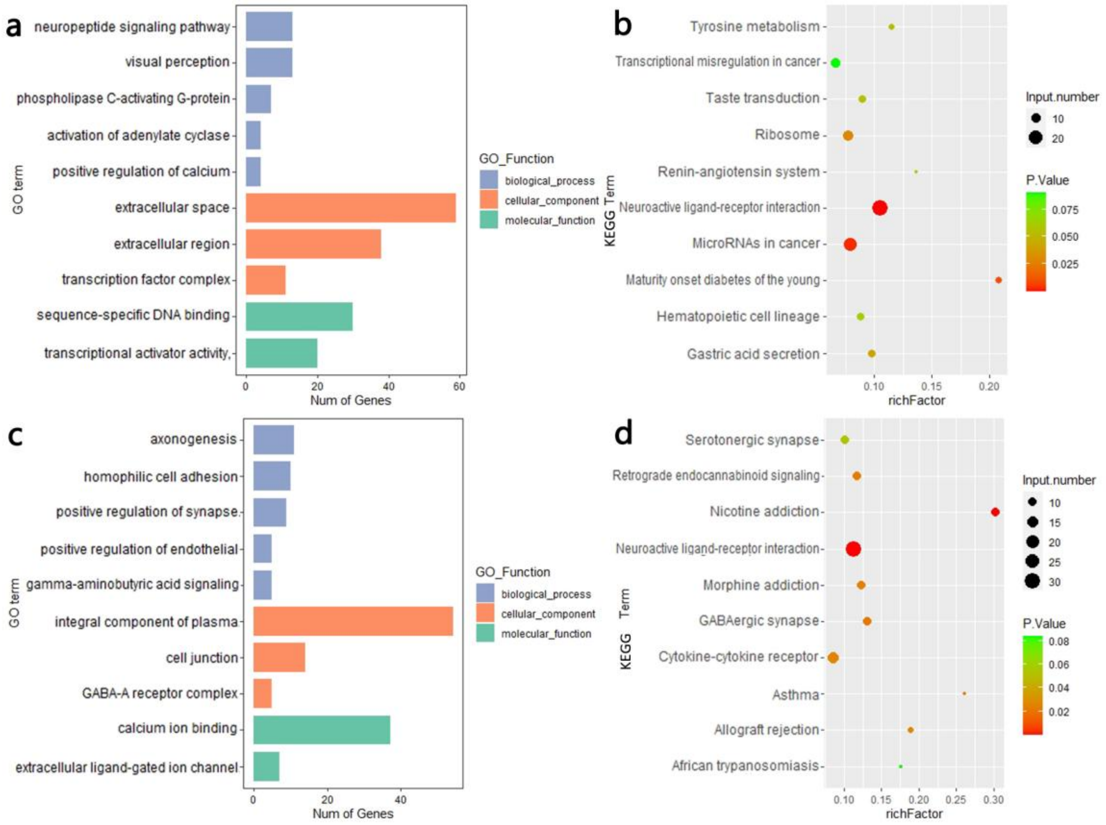

Supplement: Supplementary file 1 [file genes-12-01374-s001.zip › Figure S2.pdf]
